# Donating Health Data to Research: Influential Characteristics of Individuals Engaging in Self-Tracking

**DOI:** 10.3390/ijerph19159454

**Published:** 2022-08-02

**Authors:** Katharina Pilgrim, Sabine Bohnet-Joschko

**Affiliations:** Management and Innovation in Health Care, Faculty of Management, Economics and Society, Witten/Herdecke University, Alfred-Herrhausen-Str. 50, 58455 Witten, Germany; sabine.bohnet-joschko@uni-wh.de

**Keywords:** health self-tracking, data donation, data sharing, quantified self, mobile tracking

## Abstract

Health self-tracking is an ongoing trend as software and hardware evolve, making the collection of personal data not only fun for users but also increasingly interesting for public health research. In a quantitative approach we studied German health self-trackers (N = 919) for differences in their data disclosure behavior by comparing data showing and sharing behavior among peers and their willingness to donate data to research. In addition, we examined user characteristics that may positively influence willingness to make the self-tracked data available to research and propose a framework for structuring research related to self-measurement. Results show that users’ willingness to disclose data as a “donation” more than doubled compared to their “sharing” behavior (willingness to donate = 4.5/10; sharing frequency = 2.09/10). Younger men (up to 34 years), who record their vital signs daily, are less concerned about privacy, regularly donate money, and share their data with third parties because they want to receive feedback, are most likely to donate data to research and are thus a promising target audience for health data donation appeals. The paper adds to qualitative accounts of self-tracking but also engages with discussions around data sharing and privacy.

## 1. Introduction

The market for smartphone and smart wearable mobile applications and thus possibilities to measure, visualize or record personal health or activity related data is developing rapidly [1,2]. In 2019, 45% of German smartphone users already feature health or fitness applications on their devices [1,3]. Social change in health and lifestyle needs is driving this trend [4] as the individual self is still one of the most interesting subject domains for people to explore themselves [5]. Self-observation, self-monitoring or self-measurement generates previously unknown information serving as vehicle for facilitating action, which can be an element of empowerment, self-determination, and control [6,7,8]. Self-motivation, self-discipline, or the desire of performance enhancement are further motives to engage in self-measurement activities regarding health-related data [9,10,11,12]. While wearable devices and personal monitoring systems promote remote monitoring and data collection [13,14] privacy and security exposure is a credible threat due to limited confidentiality, integrity, authentication and access control capabilities of Internet of Things devices [15,16].

At the same time, the real world data can add value to the healthcare sector [17] by supporting pharmaceutical innovation development, accelerating rare disease diagnosis or improving chronic disease treatment [18,19,20,21,22,23,24]. Access to this data for research purposes is key in this context. Therefore, in this paper we investigate the specific user group of German health self-trackers, regarding users’ willingness to donate self-tracked data for research and compare this with their data sharing and showing behavior. Further, we try to identify sociodemographic, biometric, psychographic and behavioral characteristics possibly increasing digital data disclosure willingness for (medical) research, both by employing a digital questionnaire and multiple regression analysis. Thus, the objectives of this study are to (1) provide initial insights into the effects of different health data requests and to (2) outline specific user characteristics that seem to influence voluntary data donation willingness among active (Germany) health self-trackers for research positively. We discuss our findings with current literature and point out implications for practice.

The study contributes to a better understanding of how to frame potentially successful requests for voluntary data donation and provides valuable insights on strategies to encourage data donation by presenting actionable data describing the characteristics of active health self-trackers and those willing to donate personal self-collected health-related data for research.

## 2. Background

### 2.1. Self-Measurement Framework

In media and literature, concepts used in this context such as self-tracking, lifelogging, the quantified self, personal analytics, self-quantification, self-hacking or personal informatics are blurredly defined or used synonymously [24,25,26]. We developed a framework to generate a mutual understanding and structure related research. Going back to where it all started: The launch of the first iPhone in late 2007 established the now global Quantified Self (QS) movement started by journalists Gary Wolf and Kevin Kelly in Silicon Valley, for sharing data collection techniques as well as the collected data itself [27]. The common objective is to generate additional knowledge that preempts behavioral change [8,28]. In this context [of individual quantification], we need to define two terms: lifelogging and self-tracking. Both describe voluntary, self-directed and self-intentional monitoring as well as recording certain personal characteristics via digital technologies [24,29,30]. These also both refer to the practice of regularly collecting personal data, often related to one’s bodily functions and daily habits, followed by data analysis to generate statistics or graphs [31,32]. Specifically, Selke understands lifelogging from a sociological perspective as a generic term for capturing one’s own life in real time by recording all behavioral and data traces, storing them in a memory bank and keeping them on hand for later retrieval [30]. In addition to the categories, human tracking, human digital memory, and surveillance, Selke defines self-tracking specifically and solely as “body and health monitoring”, and a sub-aspect of lifelogging [30]. Kelly on the other hand, co-founder of the QS movement, defines lifelogging’s intent as recording and archiving whatever happens in life [33,34]. This includes all texts, visual information, sounds, media activities, and biological data collected via sensors. Archiving and the (controlled) sharing of information with peers are also decisive parameters of lifelogging. Gurrin et al. [26] follow a similar understanding in which lifelogging is a new concept utilizing wearable devices to generate a media rich archive of users’ life experience. Doherty et al. [35] summarize lifelogging as the process of automatically recording aspects of one’s life in digital form. A specific technique of lifelogging, which has drawn particular attention in research, is visual lifelogging regarding privacy issues [36,37,38,39]. This activity enables users to capture images from a first-person perspective passively via camera and ultimately creates a visual diary encoding every possible life aspect with unprecedented details [37].

Drawing from existing definitions of lifelogging in literature, we conclude that “lifelogging” refers specifically to the logging of many, or all parameters of life, and the sharing of this archived information with third parties to identify potential correlations and expand knowledge. Similar to the definition of exploratory research; lifelogging quantifies and logs life parameters without specific goals or predefined hypotheses to test, because (1) it is possible and (2) the quantified results are part of the identity.

Looking at definitions regarding “self-tracking” the Washington Post and the Wall Street Journal defined quantified self-tracking (the quantifiable self-measurement) as early as 2008 to be applied to a variety of life domains such as time management, travel, and social communication. At the same time, self-tracking also encompassed the health context with the broader definition of health as applicable to both medical issues as well as wellness goals [8,40,41]. Data generated by means of self-tracking and actions drawn upon them derive their evidential value from the (scientific) nature of their analyses [42]. In contrast to Selke’s definition, the concept of self-tacking is not to be subordinated exclusively and unambiguously to lifelogging but should be additionally equated with it. Taking a further look at self-tracking, we can differentiate between economic and health-related goals. The economic goals of self-tracking include, for example, a home’s energy consumption or personal related expenditures [43,44]. A well-defined objective is the efficiency increase of cost-burdening resource use based on tracked (economic) parameters (in order to reduce consumption and associated costs as well as to increase available personal discretionary income).

However, a review of current literature suggests a strong focus on self-tracking specifically associated with body- or health-related personal data [6,45,46,47,48]. The already established literary term of “health self-tracking” refers to the purposeful monitoring and measurement of one’s own body [6]. As self-tracking data is considered credible, objective, neutral, scientific, and trustworthy [24,49], health self-tracking is perceived as an evidence-based approach to personal improvement through changes in lifestyle [50]. With an interview-based approach regarding self-trackers data sharing behavior Lupton found, that in-app sharing options offered by apps and platforms to easily share personal data and invite the responses of other users were resisted or ignored by nearly all the members of interviewed self-trackers, who instead rather discussed results with close family members [51]. We conclude that unlike the logging of life parameters, the digital self-measurement is therefore set up and conducted in a hypothesis-testing manner because: (1) individuals pursue a specific goal and (2) the results are mainly private and not public. Since we define self-tracking as a predominantly private activity, research is eager to investigate further user motives for voluntary data disclosure.

**Figure 1:**
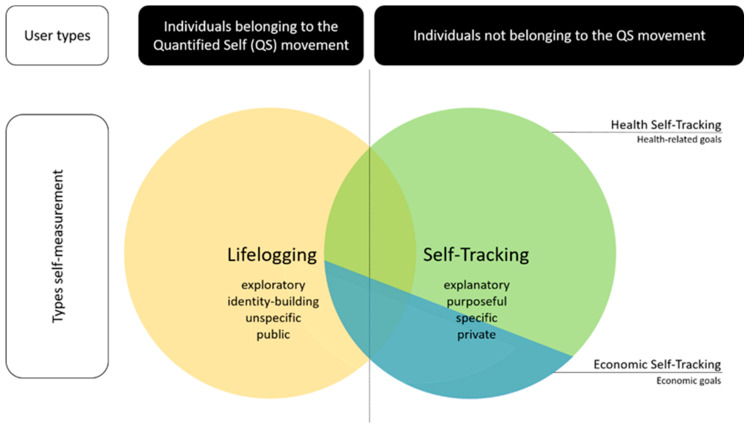
Self-measurement framework.

Earlier explicit health self-tracking cluster approaches predominately cover differentiated data recording methods and technical tools employed [1,52]. Prior to this however, systematization of health self-tracking goals is necessary. Use cases examined address testing acceptance and effectiveness of health self-tracking techniques or tools for a specific indication or target population [46,53]. Research investigates health self-tracking motives by implicit differentiation of user types, according to the Behavioral Continuum of Care Model. Hence, research investigates users separately e.g., those with existing diagnosed disease or risk factors (targeting recovery and treatment), users with self-perceived disease risk factors (targeting prevention), and users without diagnosed or self-perceived disease or prevalence (targeting wellness, fitness, or lifestyle goals, referred to as health promotion) [54,55,56,57]. Health self-trackers collectively possess a desire to use digital technologies to optimize health and well-being via self-monitoring [6,7,58]. Self-motivation, self-discipline, or the desire of performance enhancement are motives in every user group [9,10,11,12]. Figure 1 visualizes our systematization in a self-measurement framework.

### 2.2. Research Questions and Hypothesis Development

Looking at research regarding data disclosure among peers and/or on public platforms (within an app for example), cost-benefit trade-offs in this context are strongly linked to situationally perceived suffering and experience [59]. The prospects of fulfilling a feeling of belonging (to a community) and identification with the personalized and individual data can outweigh possible negatives, for example, receiving personalized advertisements or privacy concerns [60,61]. Lupton [51] states, that online patient support groups such as PatientsLikeMe as well as Facebook groups and other social media also encourage members to disclose their health, fitness and medical details as a way of contributing to peer networks of expertise and support. Disclosure can mean sharing the self-tracked data via in-app sharing features or by screenshotting results and sharing this screenshot, which in the following we label as data showing. Screenshots themselves are increasingly popular objects of analysis in new media and communications studies, and have been explored in qualitative self-tracking research, as method, as data, and as communicative practice [31,62,63].

Concerning the willingness to share personal self-tracking data with a health insurance company for example, privacy risks always have a negative effect, whereas positive effects of privacy benefits are partly dependent on data sensitivity [64]. Motives for donating personal data in general are consistent with motives supporting prosocial behavior such as blood donation [65,66,67,68]. The strongest predictor is social responsibility or a sense of duty (Skatova & Goulding, 2019). The understanding of purpose positively influences the willingness to donate data as well. In contrast, individual self-tracking motives negatively influence the willingness to donate data [69]. We ask the following questions:

RQ1: Do active health self-trackers evaluate (controlled) data donation for research differently than (uncontrolled) data sharing within the app or via screenshot across alternative platforms?

RQ2: Are there factors or characteristics concerning data sharing behavior and motives, tracked parameters and other donation behavior, that potentially influence the willingness to donate data positively?

At the same time, research on motives for engaging in health self-tracking and sharing tracked data with third parties conflicts with findings on motives for the willingness to donate user data. Although on the one hand egoistic motives have a positive influence on health-self tracking engagement and disclosing data with the community, they influence the willingness to donate data (in general) negatively on the other hand. To also address the identified opposing effects of egoistic health-self tracing motives in context of data donation behavior two hypothesis to test are:

**H1.**  *Existing egoistic motives for engaging in health-self-tracking have a negative impact on willingness to donate personal self-collected health-related data for research.*

**H2.**  *Existing egoistic motives regarding the sharing of self-tracked health-related date have a negative impact on willingness to donate this data for research.*

## 3. Materials and Methods

We used a digital questionnaire in LimeSurvey including 32 items in total. In addition to three sociodemographic parameters: (i) gender, (ii) age and (iii) education, 29 items on biometric, psychographic and behavioral characteristics were included. We started by querying (iv) weekly engagement in sport-related activity (none; up to three hours or more than three hours) and (v) devices used for tracking health-related data (smartphone, smartwatch, fitness tracker or none; multiple answers were possible) as well as (vi) frequency of accessing the tracked data (daily, weekly, monthly, less than once per month or never). Questions on tracked items were also included covering indications on tracking frequencies of: (vii) movement (distance covered), (viii) vital-parameters (e.g. pulse and blood-pressure), (ix) blood levels (oxygen saturation or glucose level), (x) hormone levels, (xi) nutrition intake (regarding macro and micro nutrients) and (xii) energy intake and consumption (calories), (xiii) specific in-app success (e.g., using fitness apps with different level programs), (xiv) sleep (duration and depth) or (xv) others. To test our hypothesis H1 we additionally queried motives for engaging in health-self tracking ((xvi) curiosity—no goal; (xvii) self-motivation and (xviii) self-monitoring).

Further items are frequency of sharing (xix) or showing (xx) data as well as (xxi) importance of privacy. Concerning hypothesis H2 we queried motives for sharing or showing tracked data (no reason (xxii), pride (xxiii), to motivate others (xxiv) and desire for feedback (xxv)). We then asked for the willingness to donate self-tracked data for research (xxvi). Questions regarding offline donation behavior include frequency of blood (xxvii), clothes (xxviii) or monetary donations (xxix) as well as volunteering (xxx). We added questions on organ (xxxi) or bone marrow donor status (xxxii). We used an 11-point scale between 0 and 10 for rating relevance and frequency of use with endpoints being descriptive rather than numerical, such as never and always or does not apply and fully applies. For hypothesis validation, we used the Spearman rank correlation coefficient and multiple regression analysis.

We collected 1091 questionnaires in January and February 2021. The recruitment strategy included digital social media channels such as Facebook, Instagram, LinkedIn, Xing, and Twitter. Facebook groups dedicated to fitness and nutrition topics, as well as Instagram stories of fitness micro-influencers, represented key channels. Defined inclusion criteria were positive statements on the items (v) devices used for tracking health-related data (smartwatch, tracker or smartphones) and (vi) frequency of accessing the tracked data (daily, weekly, monthly or less than once per month). We excluded 55 observations as participants did not use a tracking device and eight because participants never accessed the tracked data, since we want to target only active health self-trackers. In addition, we removed 109 incomplete observations. Data processing then involved encoding text format data into numeric indicator variables. For both, data preparation and analysis, we used SPSS.

## 4. Results

### 4.1. Sample

The sample (N = 919) consists of 68% women and 32% men. Overall, only two of the 919 participants did not graduate high school (0.2%). 4.5% are still in school or high school graduates. 44% are currently at, or have completed College, and 53% are currently at University or hold an University degree. 45% are between 18 and 34 years old, 46% between 35 and 54 and 9% over 55. Additionally, we know that 38% of our participants engage in up to three hours of physical sport-related activity per week. More than half (52%) exercise more than three hours a week and only 9% do not work out at all. We found that 60% of our sample uses a smartwatch for health self-tracking. 43% use a smartphone and another 33% a fitness tracker (multiple answers were possible). In terms of tracking frequency, 85% reported daily tracking. 11% track weekly, 1% tracks monthly and 2% track at a frequency lower than once per month. Reasons for health self-tracking were 52% self-motivation and 49% self-monitoring. 33% had no specific reasons but merely used tracking out of curiosity (multiple answers were possible).

Overall, 96% track movement parameters, such as number of steps or distance covered. 66% tracked vital signs, 56% calories, 55% sleep patterns, 32% nutrition, 22% hormones and 10% blood values. Another 32% track the progress of app-based fitness programs and 12% track other parameters not covered in the questionnaire. Multiple answers were also possible. Privacy and data security play no role at all for 3% of the sample, and are very important for 32%. The average importance is 7.2 (out of 10). Considering other donation activities from irregularly to regularly, we found that 42% donate blood, 94% donate clothes, 77% donate money, and 45% volunteer. At the same time, 57% hold an organ donor card. In this declaration of will in the event of death, the issuing person declares whether he or she agrees to donate all or some organs and tissues or whether he or she rejects their removal. In our case, we specifically asked for consent to organ donation in general. We yet simplified organ donation to a yes/no status for the purposes of the survey (rather than considering organ-by-organ donation preferences). Finally, 49% are registered bone marrow donors.

41% share their tracked data sometimes to always. 49% show their results via screenshot in another app such as Instagram or Facebook sometimes to always. When asked why users either share or show their results, 51% indicate pride. 39% share or show their data because they want feedback, 44% want to motivate others with their results and 31% have no specific reason for doing so or have never thought about it. Multiple answers were also possible here. 22% of respondents would not donate their data under any circumstances. 26% state the probability of donating their data as 10% to 40% and can thus be categorized as rather unwilling to donate data. 11% of all respondents are undecided and place their probability of willingness to donate at 50%. Approximately one-third (31%) are more likely to donate data, ranging between 60 and 90%. Finally, 10% of health self-trackers surveyed indicate they are 100% likely to donate their data for research.

### 4.2. Data Sharing and Showing Behaviors Compared to Data Donation Willingness

To address our first question, we compared responses to the questions on willingness to donate tracked data (with a range 0 = not at all to 10 = definitely) and on (in app) data sharing or data showing (via screenshot within other platforms) behavior (with a range 0 = never to 10 = always) (Table 1). A mean comparison shows that the (hypothetical) willingness to donate data (4.5 out of 10) is more than twice as high as the existing willingness to share (2.09 out of 10) or show (1.94 out of 10) data.

### 4.3. User Characteristics Influencing Data Donation Willingness

To identify potential influences on the willingness to donate, we first calculated correlations according to Spearman. We found that the following 12 variables correlate significantly either positive or negative with the willingness to donate: (i) age, (ii) gender, (iii) tracking frequency, (iv) tracking vital signs, (v) data sharing behavior, (vi) data showing behavior, (vii & viii) reasons for tracking: self-motivation and self-monitoring, (ix & x) reasons for sharing or showing: feedback and to motivate others, (xi) importance of privacy and (xii) monetary donating behavior. Except for monetary data donation behavior, there is no correlation between other donation behavior or willingness (clothes, blood, organs, etc.) and the willingness to donate data. We then tested a multiple regression model of correlating variables to determine the characteristics among health self-trackers contributing to data donation. Our final model can explain a total of 13.7% of the variance and ultimately includes the following nine variables after we removed three strongly correlated variables to improve the model: (i) age, (ii) gender, (iii) tracking frequency, (iv) tracking vital signs, (v) data sharing behavior, (vi) data showing behavior, (vii) reason for sharing or showing: feedback, (viii) importance of privacy and (ix) monetary donating behavior.

Overall, all significantly correlating parameters also have a significant influence on the willingness to donate (Table 2). Two parameters have a negative influence: the higher the relevance of privacy (β coefficient= −1.09; *p* = 0.009) or the older (β coefficient= −3.998; *p* = 0.25), the lower the willingness of data donation. Other parameters increase the probability to donate self-tracked data. Observed effects are significant with a relatively small effect.

We further investigated whether egoistic motives for health-self-tracking or data sharing have a negative impact on data donation willingness (H1 and H2). Results can not confirm hypothesis 1—in contrast, individuals who indicated self-motivation or self-monitoring as health self-tracking motives had a greater willingness to donate compared to users tracking with no goal. The influence of these egoistic motives on the willingness to donate is positive and weakly significant (β coefficient = 5.751; *p* = 0.028 and β coefficient = 5.230; *p* = 0.045) (Table 3). Also, two out of three egoistic motives for in-app data sharing, (i) the desire for feedback and (ii) to motivate others, have a significant positive impact on the willingness to donate data, thus we can not confirm hypothesis H2 either (β coefficient = 2.008; *p* < 0.001 and β coefficient = 1.462; *p* = 0.002) (Table 4).

## 5. Discussion

Findings on individual data sharing or showing portray an interesting picture: 58% state they never show or share their data even though the research is drawing responses from a space where sharing is highlighted since social platforms are at their core, about sharing. On the one hand, these results support findings and add to our framework on self-measurement, stating that especially people that are not considered lifeloggers but health self-trackers, find this a rather private than public matter [51]. On the other hand, we argue that these statements, or perceptions, are potentially at odds with the actual data transfer within the applications. The business model of commercial providers offering applications free of charge is (often) based on the exploitation of collected user data—either to optimize their own products or to monetize data through sales. At the very least, it is reasonable to suppose that users are not the only ones with local access to their data, having confirmed consciously or unconsciously such access by downloading the app and agreeing to the General Terms of Use. Thus, we can assume that the actual relative share of health self-trackers never sharing their data is substantially lower. In reality, users are most likely to share their data continuously with the app provider (at least). Based on our results, one could argue now that a proactive request to share data with the provider during the user journey significantly lowers the willingness and therefore a “hidden” request is an attractive option for data donation as well. However, if you look at successful health data platforms like 23andMe, you find a real world example how a transparent request for allowing aggregated, de-identified customer data to be used for research is highly successful with over 80% of 10 million consenting users. Arguably, there is a different form of predictive health data at play here, compared to the common metrics of self-tracking. In addition, Harris et al. [70] argue that the notion of gift exchange is used to draw attention away from the free, clinical labor which drives the profitability of 23andMe.

Comparing answers on the willingness to donate data and data sharing and showing behavior, we clearly demonstrate, that the framing question “donating data for research” has a much higher impact than the intrinsic motive for feedback, which is stated as the main reason for in-app data sharing. Results support research by Meadows et al. [50] that people rather track their data for personal improvement through changes in lifestyle than using the data for self-presentation. We argue, that an embedded query regarding additional data donation for research could foster trust and therefore, be key to accessing self-tracking data for researchers. In contrast to already existing third party platforms or apps solely for data donation, an imbedded query could overcome data access barriers as general user convenience is taken into account. To realize this hypothetical in-app request, providers must understand the benefits of collaborating with research institutions (at no additional costs) due to increasing brand/company trust as well as perceived integrity by transparent embedded data donation request.

Further, we were able to identify a specific user type who is more likely to donate data than the rest: younger men up to 34, tracking vital signs daily, regularly donating money, and sharing or showing their data to third parties motivated by a desire for feedback are most likely to donate data to research. According to research by Karkar et al. [12] primarily individuals with existing medical conditions track vital signs or bio parameters. Here, the primary motivation of better disease management by improving personal disease knowledge and monitoring health indicators (such as glucose or blood pressure levels) are key. The underlying desire for feedback from the community as significant motivator to share data with peers could also signal a current medical condition, people are trying to shed light on with the support of the crowd. Accordingly, we argue that the user-type most likely to donate is not just male, but could also fit in the prior defined health self-tracker group of individuals with existing diagnosed disease or risk factors (targeting recovery and treatment) [56]. Looking at the recruiting period, which was January and February 2021, a global winter peak of the COVID-19 pandemic, it is thus possible, that people were especially eager to shed light on their COVID or post/long COVID symptoms by sharing their self-tracked vital parameters (among others) with the community. Recent literature suggest a growing pool of self-reported symptoms and related personal health parameters, being shared via social media or online support platforms [71,72,73]. Since situational perceived pressure of suffering can outweigh privacy concerns, individuals who aim to reduce their personal suffering are thus more likely to donate sensitive health data for research in return for the prospect of a better therapy option in the future. Indirect reciprocity can explain this behavior: giving back (to the community), expecting the same treatment in return [59,60,61]. This could imply that apps specifically for disease management offer a promising first gateway for implementing data donation requests.

Findings by Skatova and Goulding (2019), according to which (personal) motives have a negative impact on the willingness to donate personal data, are contradicted by this study’s results [65,69]. A possible reason could be the different sample structure. Skatova and Goulding surveyed a population cross-section, whereas we restricted ourselves specifically to health self-trackers, i.e., people who de facto actively track and access their health-related data. Thus, our results on the willingness to donate data confirm studies on the willingness to share data, according to which egoistic motives influence the willingness to share positively. This could suggest that additional data donation requests should be implemented in apps with a broad in-app community based on sharing personal data to compare with others preferably. Optionally, self-trackers who did share data in-app at least once could also be actively approached to donate their data right after or before they have shared data within the app.

The findings have a number of limitations. Due to our recruitment strategy, primarily conducted via social media, the given sample includes a disproportionately large number of young as well as higher educated individuals and a majority of women compared to men (2/3 to 1/3) considered a sample bias. In addition, a maximum of just under 14 percent of the variance regarding the probability of data donation can be explained via multiple regression in our model. This indicates the parameters queried in the questionnaire, i.e., the selected model, fail to take into account factors that possibly have a much greater influence. As income (for adults over 55) is a known influence on the willingness to donate data for science positively [74], which we did not capture, future studies may therefore repeat the survey with a larger sample via additional recruitment channels, adding income as demographic variables to not only assess the reliability of our findings but possibly generate an even better model. Further, in our study we did not address aspects regarding “perceived usefulness of data donation” or “personal benefits from data donation” which could potentially give better insights concerning the motivation to donate data as well as how to address persons so that they are willing to donate their data. Future studies could follow up on these additional factors with a qualitative approach.

## 6. Conclusions

Reviewing current literature on self-measurement, we were able to outline a conceptual framework, differentiating the terms Quantified Self, lifelogging, self-tracking and health-self tracking. The framework outlined differences and similarities and can therefore foster orientation and structure for future research. Our quantitative results provide initial insights into the effects of different health data requests and contribute to a better understanding of how potentially successful requests for voluntary data donation are framed. When requests for altruistic data donation for research are framed as such, willingness is more than twice as high compared to actual in-app sharing behavior or frequency of sharing personal health data with third parties via screenshot.

We were also able to outline, that specific user characteristics seem to influence data donation willingness positively: if the user is male, under 34, tracks vital-parameters daily and shares or shows his data motivated by the desire for feedback. Personal motives for self-tracking or sharing the data do not influence data-donation willingness negatively; therefore described health self-trackers are a promising target group for data donation requests. Our findings could help future efforts to approach health self-trackers for data donation to support research. Collaborations in this context between research institutes and commercial self-tracking application providers could benefit both parties in the end.

## Figures and Tables

**Table 1:** Comparison of data donation willingness, data sharing and data showing behavior.

		Probability to Donate	Sharing Results	Showing Results
**N**	**Valid**	**919**	**919**	**919**
	Missing	0	0	0
Mean		4.51	2.09	1.94
Median		4.60 a	0.64 a	0.80 a
Std. Deviation		3.542	3.195	2.662
Variance		12.549	10.209	7.088
Skewness		0.087	1.374	1.320
Std. Error of Skewness		0.081	0.081	0.081
Kurtosis		−1.431	0.509	0.737
Std. Error of Kurtosis		0.161	0.161	0.161
Range		10	10	10
Minimum		0	0	0
Maximum		10	10	10
Percentiles	25	0.92 b	.b,c	.b,c
	50	4.60	0.64	0.80
	75	7.73	3.54	3.40

a. Calculated from grouped data. b. Percentiles are calculated from grouped data. c. The lower bound of the first interval or the upper bound of the last interval is not known. Some percentiles are undefined.

**Table 2:** Influencing parameters on data donation probability.

	Unstandardized Coefficients		Standardized Coefficients		
	B	Std. Error	Beta	t	Sig.
(Constant)	27.892	6.436		4.333	0.000
Frequency of tracking	4.459	1.985	0.072	2.246	0.025
Vital-parameter tracking	5.864	2.430	0.078	2.413	0.016
Sharing results	1.282	0.459	0.116	2.791	0.005
Showing results	1.338	0.581	0.101	2.302	0.022
Reason: Wanting Feedback	1.558	0.465	0.124	3.352	0.001
Relevancy of privacy	−1.090	0.419	−0.082	−2.600	0.009
donating money	1.191	0.320	0.116	3.715	0.000
Sex	6.921	2.390	0.091	2.896	0.004
Age	−3.988	1.782	−0.073	−2.238	0.025

**Table 3:** Multiple regression to test the influences of egoistic motives for engaging in health self-tracking on the probability to donate.

	Unstandardized Coefficients		Standardized Coefficients		
Model	B	Std. Error	Beta	t	Sig.
(Constant)	40.094	2.983		13.441	0.000
Curiosity—no goal	−1.512	2.880	−0.020	−0.525	0.600
Self-Motivation	5.751	2.608	0.081	2.205	0.028
Self-Monitoring	5.230	2.603	0.074	2.009	0.045

**Table 4:** Multiple regression to test the influence of egoistic motives for sharing health-related self-tracked data on the probability to donate.

Model	Unstandardized Coefficients		Standardized Coefficients	t	Sig.
	B	Std. Error	Beta		
(Constant)	35.482	1.584		22.398	0.000
reason: proud	0.049	0.447	0.005	0.110	0.913
reason: desire for feedback	2.008	0.516	0.160	3.890	0.000
reason: to motivate others	1.462	0.467	0.149	3.130	0.002
no reason	0.868	0.376	0.075	2.309	0.021

## Data Availability

The data set can be requested from the authors with a rationale.
